# Cancer–Osteoblast Interaction Reduces *Sost* Expression in Osteoblasts and Up-Regulates lncRNA *MALAT1* in Prostate Cancer

**DOI:** 10.3390/microarrays4040503

**Published:** 2015-10-29

**Authors:** Aimy Sebastian, Nicholas R. Hum, Bryan D. Hudson, Gabriela G. Loots

**Affiliations:** 1 Lawrence Livermore National Laboratories, Physical and Life Sciences Directorate, Livermore, CA 94550, USA; E-Mails: asebastian2@ucmerced.edu (A.S.); hum3@llnl.gov (N.R.H); bryandhudson@gmail.com (B.D.H.); 2 School of Natural Sciences, University of California, Merced, CA 95343, USA

**Keywords:** prostate cancer, osteoblast, *MALAT1*, *Sost*, co-culture, *Mmp13*, *Il6*, *Tgfb2*, PC3

## Abstract

Dynamic interaction between prostate cancer and the bone microenvironment is a major contributor to metastasis of prostate cancer to bone. In this study, we utilized an *in vitro* co-culture model of PC3 prostate cancer cells and osteoblasts followed by microarray based gene expression profiling to identify previously unrecognized prostate cancer–bone microenvironment interactions. Factors secreted by PC3 cells resulted in the up-regulation of many genes in osteoblasts associated with bone metabolism and cancer metastasis, including *Mmp13*, *Il*-*6* and *Tgfb2*, and down-regulation of Wnt inhibitor *Sost*. To determine whether altered *Sost* expression in the bone microenvironment has an effect on prostate cancer metastasis, we co-cultured PC3 cells with Sost knockout (*Sost*^KO^) osteoblasts and wildtype (WT) osteoblasts and identified several genes differentially regulated between PC3-*Sost*^KO^ osteoblast co-cultures and PC3-WT osteoblast co-cultures. Co-culturing PC3 cells with WT osteoblasts up-regulated cancer-associated long noncoding RNA (lncRNA) *MALAT1* in PC3 cells. *MALAT1* expression was further enhanced when PC3 cells were co-cultured with Sost^KO^ osteoblasts and treatment with recombinant Sost down-regulated *MALAT1* expression in these cells. Our results suggest that reduced *Sost* expression in the tumor microenvironment may promote bone metastasis by up-regulating *MALAT1* in prostate cancer.

## 1. Introduction

Prostate cancer is the most frequent form of cancer in males and a leading cause of cancer death among men of all races [1]. Bone metastasis is very common in patients with advanced prostate cancer and is associated with high mortality and morbidity [2,3]. Cancer metastasis is a complex process where the cancer cells detach from the primary site, undergo epithelial–mesenchymal transition, travel through the circulatory or lymphatic systems, and form secondary tumors [2,3]. It has been hypothesized that the bone microenvironment serves as a rich “soil” by secreting factors that promote survival and propagation of cancer cells [2,3]; in turn tumors secrete factors that alter the bone microenvironment to promote metastatic colonization [2,3]. Multiple factors involved in bone metastasis, including growth factors, cytokines and matrix metalloproteinases [3,4,5], have already been identified, however, the mechanisms responsible for bone metastasis are not yet fully understood. Development of new therapies for the prevention and treatment of prostate cancer bone metastasis depends on understanding the dynamic reciprocal interactions between prostate cancer and the bone microenvironment.

Osteoblasts are bone forming cells of mesenchymal origin that are responsible for the synthesis and mineralization of bone matrix [6]. Prostate cancer cells interact with mesenchymal-derived tissue at the site of metastasis, which subsequently alters the bone microenvironment and contributes to the malignant progression of prostate cancer [4]. Many studies have previously investigated cancer–osteoblast interactions in the context of bone metastasis [7,8,9]. A number of such studies have used *in vitro* co-culture models [8,9,10] to explore osteoblast-tumor cell interactions and successfully identified numerous factors that contribute to bone metastasis [5,8,9,10]. However, it is likely that there are other key molecules that remain unidentified.

Gene expression profiling using microarrays has proven effective in studying changes of large sets of transcripts, simultaneously. In this study, we investigated prostate cancer–osteoblast interactions using microarrays to identify novel factors that contribute to prostate cancer metastasis to bone. We co-cultured highly invasive PC3 cells with either immortalized or primary osteoblasts and profiled gene expression changes. Gene expression data analysis identified several genes, including *Il6*, *Tgfb2*, *Cxcl1*, *Mmp13*, *Ctgf*, *Sost* and lncRNA *MALAT1*, differentially regulated in co-cultures compared to monocultures. We also identified *Sost* as a regulator of *MALAT1* in PC3 cells. This study provides novel insights into cellular responses in relation to prostate cancer–osteoblast interactions with potential therapeutic implications.

## 2. Experimental Section

### 2.1. Animals

Wildtype C57BL/6 (WT) and *Sost*^KO^ mice (C57BL/6 background) [11] were used in this study. All animal experiments were approved by the Lawrence Livermore National Laboratory Institutional Animal Care and Use Committee and followed the U.S. National Institutes of Health “Using Animals in Intramural Research” guidelines for animal use.

### 2.2. Cell Culture

Mouse primary osteoblasts (OBs) were collected similar to Bellows *et al.* 1986 [12], with a few adjustments. Briefly, calvaria from 4–5 day old WT and *Sost*^KO^ mice were dissected aseptically by stripping the frontal and parietal bones of periosteum and loosely adherent tissue, cut into small pieces, and sequentially digested 3 times at 37 °C in 5 mL of collagenase solution (Collagenase 1, 2.4 mg/mL in DMEM/F-12), followed by alternating 5 mM EDTA and Collagenase digestions for 3 subsequent digestions. Isolated OBs from fractions 3–6 were seeded at 4 × 10^4^ cells/cm^2^ at 37 °C in 4 mL of collagenase solution (Collagenase 1, 0.625 mg/mL; Collagenase B, 1.875 mg/mL; CaCl2, 25 mM; in ddH20 on ice) mixed 1:2.5 with media solution (DMEM/F-12, 0.1% BSA, 25 mM Hepes, 37 °C). PC3 cells (ATCC, Manassas, VA, USA), UMR-106 cells (ATCC) and primary osteoblasts were cultured in either a 12 well plate or a 3.0 µm pore transwell insert until cells reached 50% confluency in DMEM/F-12 media supplemented with 10% fetal bovine serum and 1% Penicillin/Streptomycin. Media was changed to serum-free DMEM/F-12 media prior to addition of recombinant human SOST (R & D Systems, Minneapolis, MN, USA ) at 100 ng/mL, then cultured for 48 h prior to RNA isolation.

### 2.3. Microarrays

PC3 cells and UMR-106 cells cultured under mono- or co-culture conditions for 48 h were isolated by centrifugation and total RNA was extracted using an RNeasy Mini Kit (QIAGEN, Valencia, CA, USA, according to the manufacturer’s guidelines. Samples were biotin labeled and hybridized on Human Genome U133 Plus 2.0 oligonucleotide array (PC3) and Rat Genome 230 2.0 Array (UMR-106) (Affymetrix, Santa Clara, CA, USA), according to the manufacturer’s recommendations. Microarray data analysis was conducted using Bioconductor [13]. Data preprocessing and normalization were performed using Robust Multi-chip Average (RMA) protocol [14]. Subsequently, we filtered the data using the nsfilter function from the genefilter package on bioconductor to remove invariant transcripts and to exclude Affymetrix control probes. Genes differentially expressed between different conditions were identified using the empirical Bayes method implemented in Linear Models for MicroArray (LIMMA) package [15]. Genes with an FDR corrected *p*-value less than 0.05 and fold change greater than 2 were considered significantly differentially expressed. Heat map analysis was conducted using the “heatmap.2” function from the R package gplots. The raw microarray data have been deposited in the Gene Expression Omnibus (GEO accession number: GSE73044).

We also obtained metastatic and primary prostate cancer patient gene expression data from GEO (GSE3325) and identified genes differentially expressed between metastatic prostate cancer and clinically localized primary prostate cancer as described above. Differentially expressed genes identified in the PC3-UMR co-culture assay were subsequently compared to genes differentially expressed in metastatic prostate cancer patient samples and potential candidate genes associated with prostate cancer metastasis that are also inducible by factors secreted by osteoblasts were identified.

### 2.4. Functional Annotation

Gene Ontology analysis was performed using ToppGene Suite [16]. GO terms with FDR corrected *p*-value < 0.05 were considered enriched. Protein interactions maps for differentially expressed genes were generated using GeneMANIA [17] and Cytoscape [18].

### 2.5. Immunocytochemistry

UMR-106 cells and PC3 cells were grown in DMEM/F-12 media supplemented with 10% fetal bovine serum and 1% Penicillin/Streptomycin until the cells reached 50% confluency. Subsequently, the cells were added to transwell inserts with 3 µm pore size and the media was changed to serum free media. Cultures were incubated for 48 h prior to fixation with 4% paraformaldehyde. Cell membranes were permeabilized with 0.1% Triton X-100 prior to blocking in PBST + 10% fetal bovine serum and staining with 5 µg/mL of Sost antibody (AF1589, R & D Systems) in blocking buffer for 1 h. Subsequent secondary antibody staining was performed for 1 h with Alexa Fluor^®^ 594 goat anti-Mouse Secondary Antibody followed by DAPI staining. Imaging was performed using a Leica DM50000B.

### 2.6. Quantitative PCR

Total RNA was purified using RNeasy Mini Kit (QIAGEN, Valencia, CA, USA) according to manufacturer’s protocol. Superscript III First-Strand Synthesis System (Invitrogen, Grand Island, NY, USA) was used with random hexamer primers for reverse transcription according to manufacturer’s protocol. Real time quantitative PCR was then performed with SYBR Select Master Mix (Applied Biosystems, Grand Island, NY, USA) using a Applied Biosystems 7900HT Fast Real-Time PCR System with the following cycling conditions: 50 °C for 2 min for SYBR then 95 °C for 3 min (2 min for SYBR), followed by 40 cycles of 95 °C for 3 s (10 s for SYBR) and 30 s at 60 °C. Data were normalized to control genes (GAPDH) and fold changes were calculated using the comparative *C*_T_ method [19]. Three independent replicates were analyzed per condition. Primers used for quantitative PCR (qPCR) are given in Supplementary Table 1 (Table S1).

## 3. Results

### 3.1. Molecular Changes in Osteoblasts Co-Cultured with Prostate Cancer Cells

**Figure 1:**
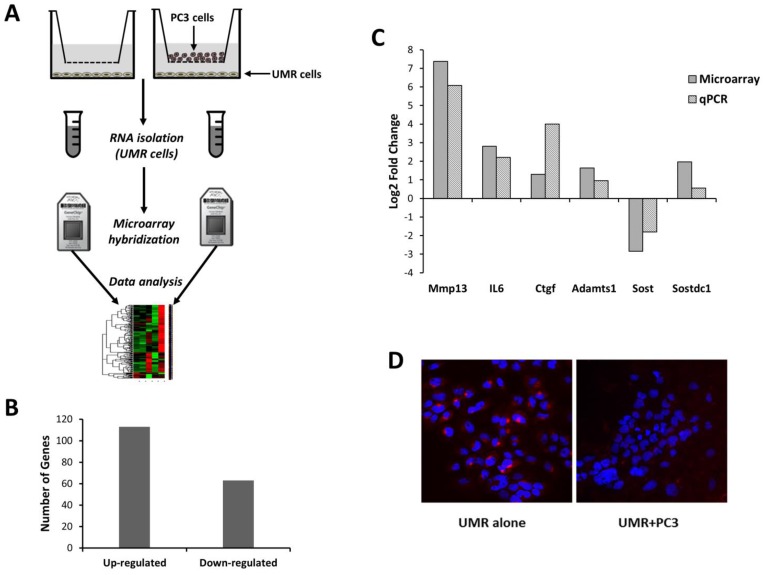
Co-culture of UMR-106 osteoblastic cells with prostate cancer cells (PC3) promotes changes in gene expression. (**A**) UMR-106 cells were cultured alone or with PC3 cells in transwells, and gene expression changes were quantified using microarrays; (**B**) 113 and 63 genes were found differentially expressed between UMR cells co-cultured with PC3 cells compared to UMR cells alone; (**C**) a subset of these differentially transcribed genes were confirmed using qPCR; and (**D**) immunocytochemistry showed a reduction in Sost protein expression in UMR cells co-cultured with PC3 cells (UMR + PC3) compared to UMR cells cultured alone (UMR alone).

To understand how factors secreted by prostate cancer cells regulate gene expression in osteoblasts we co-cultured rat osteosarcoma derived osteoblastic cells (UMR-106) with an osteolytic prostate cancer cell line (PC3) on transwell plates without physical contact (Figure 1A). UMR cells were also cultured alone as the control. By comparing gene expression in osteoblast co-cultures to monocultures we identified 113 up- and 63 down-regulated genes (Figure 1B, Table S2). Differentially expressed genes included several known regulators of bone metabolism, such as *Gpnmb* [20], *Fhl2* [21], *Mgp* [22], *Enpp1* [23] and *Phex* [24].

Factors secreted by osteoblasts have been shown to play a major role in regulating cancer metastasis to bone [25,26]. This prompted us to investigate the changes in the regulation of the osteoblast secretome in response to osteoblast-prostate cancer interactions. Our analysis identified 41 genes encoding secreted proteins (Table 1) that are more than two-fold up- or down-regulated in osteoblast co-cultures compared to monocultures. We also found Wnt pathway inhibitors *Sost* [27] and its paralog *Sostdc1* [28] with altered expression in co-cultured osteoblasts. *Sost* expression was significantly down-regulated, while its paralog *Sostdc1* was significantly up-regulated in co-cultured osteoblasts (Table 1). Using qPCR we confirmed the differential expression of a subset of genes, including *Mmp13*, *Ctgf*, *Il-6*, *Adamts1*, *Sost* and *Sostdc1* (Figure 1C), in osteoblast co-cultures.

**Table 1:** Genes encoding secreted proteins more than two-fold up- or down-regulated in UMR-106 osteoblastic cells co-cultured with PC3 cells compared to UMR cells cultured alone.

Gene	Log2 Fold Change	Adjusted *p*-Value
*Mmp13*	7.379	0.00268
*Lbp*	4.111	0.00578
*Wisp2*	3.857	0.01510
*Slpi*	3.636	0.00614
*A2m*	3.472	0.01160
*Cp*	3.436	0.01553
*Il1rl1*	3.408	0.01955
*Igfbp5*	3.005	0.01046
*Il6*	2.808	0.01311
*Cxcl1*	2.551	0.01750
*Mgp*	2.518	0.03807
*Fam20c*	2.289	0.01510
*Sfrp4*	2.284	0.01831
*Fst*	2.161	0.03814
*Adamts4*	2.044	0.03251
*Sostdc1*	1.968	0.01953
*Lum*	1.745	0.01986
*Enpp3*	1.745	0.03105
*Sned1*	1.733	0.03146
*Adamts1*	1.64	0.03251
*Gpx3*	1.587	0.02450
*Adamts5*	1.515	0.03793
*Fndc1*	1.454	0.03494
*Igfbp4*	1.452	0.03072
*Col3a1*	1.435	0.03793
*Col14a1*	1.427	0.04376
*Clec11a*	1.425	0.03089
*Ecm1*	1.352	0.04301
*Enpp1*	1.328	0.03564
*C1qtnf1*	1.308	0.04556
*Ctgf*	1.30	0.03807
*Tgfb2*	1.194	0.04545
*Cgref1*	−1.174	0.04376
*Pcsk6*	−1.485	0.03793
*Apln*	−1.563	0.02821
*Bmp3*	−1.722	0.02335
*Cd55*	−1.738	0.02369
*Metrnl*	−1.849	0.01974
*Bmper*	−2.128	0.01553
*Sost*	−2.83	0.01510
*Mamdc2*	−2.859	0.01953

### 3.2. Functional Analysis of Differentially Regulated Genes

A gene ontology (GO) analysis of differentially expressed genes was performed using ToppGene Suite [16]; “response to hormone”, “extracellular matrix organization”, “cell migration”, “ossification” and “vasculature development” were identified to be a few of the most enriched biological processes. The top 200 enriched biological processes are listed in Table S3, while most relevant GO terms and associated genes are listed in Table 2. Of all the differentially expressed genes, secreted signaling proteins *Tgfb2*, *Il-6*, *Cxcl1*, and *Ctgf* and metallopeptidases *Mmp13*, *Adamts4*, and *Adamts5* were of particular interest, as these genes have previously been shown to play a role in regulating cancer migration and invasion [29,30,31,32,33,34,35,36] and bone remodeling [37,38,39,40,41].

**Table 2:** Enriched gene ontology terms associated with genes differentially expressed between UMR cells co-cultured with PC3 cells and UMR monocultures.

GO (Gene Ontology) ID	GO Term	Genes
GO:0001503	Ossification	*Sost*, *Col13a1*, *Cebpb*, *Cebpd* ,*Dhrs3*, *Enpp1*, *Ifitm1*, *Ecm1*, *Sbno2*, *Tgfb2*, *Phex*, *Bmp3*, *Junb*, *Fam20c*, *Igfbp5*, *Mgp*, *Phospho1*, *Egr2*, *Jag1*, *Ctgf*, *Gpnmb*, *Mmp13*, *Fhl2*, *Csgalnact1*, *Il6*
GO:0009725	Response to hormone	*Hhex*, *Sost*, *Sfrp4*, *Rarb*, *Enpp1*, *Fos*, *Dusp1*, *Gatm*, *Cd55*, *Irf1*, *Cav1*, *Nr4a1*, *Stat3*, *Tgfb2*, *Cryab*, *Junb*, *Btg2*, *Egr1*, *Egr2*, *Ass1*, *Kcnma1*, *Prkar1b*, *Ctgf*, *Fhl2*, *Gng5*, *Il6*
GO:0030198	Extracellular matrix organization	*Col3a1*, *A2m*, *Col13a1*, *Adamts4*, *Pdgfra*, *Pecam1*, *Adamts5*, *Itgb3*, *Tgfb2*, *Col14a1*, *Ctgf*, *Lum*, *Mmp13*, *Csgalnact1*
GO:0016477	Cell migration	*Col3a1*, *Pde4b*, *Ddx58*, *Pdgfra*, *Pecam1*, *Ifitm1*, *Cav1*, *Lbp*, *Nr4a1*, *Ecm1*, *Cxcl1*, *Adarb1*, *Itgb3*, *Stat3*, *Ednrb*, *Tgfb2*, *Thbd*, *Igfbp5*, *Jag1*, *Ctgf*, *Bmper*, *Nexn*, *Il6*
GO:0045595	Regulation of cell differentiation	*Col3a1*, *Sox6*, *Sfrp4*, *Rarb*, *Cebpb*, *Cebpd*, *Pdgfra*, *Metrnl*, *Enpp1*, *Fos*, *Ntrk1* ,*Dusp6*, *Tmem176a*, *Ifitm1*, *Sostdc1*, *Irf1* , *Irx3* ,*Cav1*, *Itgb3*, *Zfp36*, *Stat3*, *Ednrb*, *Tgfb2*, *Junb*, *Fam20c*, *Bcl11b*, *Igfbp5*, *Jag1*, *Errfi1*, *Col14a1*, *Mafb*, *Ctgf*, *Maff*, *Tesc*, *Fst*, *Il6*, *Maf*
GO:0008283	Cell proliferation	*Hhex*, *Sfrp4*, *Rarb*, *Cebpb*, *Pdgfra*, *Adamts1*, *Ntrk1*, *Cd55*, *Etv4*, *Ifitm1*, *Irf1*, *Cav1*, *Nr4a1*, *Ecm1*, *Cxcl1*, *Adarb1*, *Itgb3*, *Stat3*, *Ednrb*, *Tgfb2*, *Wisp2*, *Tbx18*, *Slfn3*, *Junb*, *Bcl11b*, *Btg2*, *Apln*, *Igfbp4*, *Igfbp5*, *Egr1*, *Ifitm3*, *Cgref1*, *Clec11a*, *Jag1*, *Prkar1b*, *Ctgf*, *Il1rl1*, *Tesc*, *Gpnmb*, *Bmper*, *Fst*, *Csgalnact1*, *Il6*
GO:0001944	Vasculature development	*Col3a1*, *Hhex*, *Sfrp4*, *Ddah1*, *Pdgfra*, *Adamts1*, *Ntrk1*, *Pecam1*, *Cav1*, *Nr4a1*, *Ecm1*, *Itgb3*, *Tgfb2*, *Junb*, *Zfp36l1*, *Egr1*, *Jag1*, *Errfi1*, *Ctgf*, *Bmper*, *Il6*

**Figure 2:**
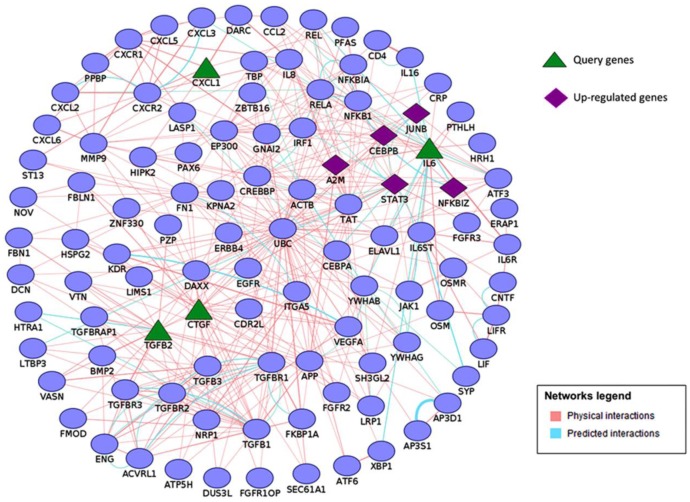
An integrated network of *Tgfb2*, *Il*-*6*, *Cxcl1*, and *Ctgf* interactions generated using GeneMANIA and Cytoscape. Query genes (triangles) and up-regulated genes (diamonds) are highlighted.

To better understand how secreted signaling proteins *Tgfb2*, *Il-6*, *Cxcl1*, and *Ctgf* may be involved in potentially regulating a complex biological process such as bone metastasis, we examined these genes in the context of protein interaction networks. GeneMANIA [17] and Cytoscape [18] were used to generate and visualize protein interactions between these genes and related genes in the network. This interaction data included physical and predicted protein–protein interactions. An integrated network of *Tgfb2*, *Il-6*, *Cxcl1*, and *Ctgf* interactions revealed several putative binding partners in osteoblasts and/or prostate cancer and suggested that these secreted cytokines may control up-regulated transcription factors *Junb*, *Cebpb* and *Stat3* in osteoblasts (Figure 2). These transcription factors have been shown to play a major role in regulating bone metabolism [42,43,44,45]. This analysis also revealed several other up-regulated genes such as *A2m* and *Nfkbiz* as members of *Il-6*, *Cxcl1*, *Ctgf*, and *Tgfb2* interactome (Figure 2). Future experimental studies may validate the potential role up-regulation of *Il-6*, *Cxcl1*, *Ctgf*, and *Tgfb2* may have in promoting cancer cell migration, invasion, and cancer-induced bone metabolism.

Metallopeptidases *Mmp13*, *Adamts4*, and *Adamts5* are associated with the enriched gene ontology term “extra cellular matrix organization”. These metallopeptidases regulate bone remodeling by degrading components of the extracellular matrix, particularly the collagens and aggrecans [36,41,46]. We found *Mmp13* to increase greater than 60 fold in osteoblast co-cultures compared to monocultures (Figure 1C). *Mmp13* has also been shown to regulate cancer-induced osteolysis [47,48,49]. *Adamts4* and *Adamts5* have been shown to promote cell growth and invasion [36]. We also identified another up-regulated Adam family member, *Adamts1*, in osteoblast co-cultures and confirmed the differential regulation of this transcript by qPCR (Figure 1C). Up-regulation of these metallopeptidases may enhance bone remodeling and promote prostate cancer invasion and growth [36,48].

### 3.3. Effect of Bone Microenvironment Derived *Sost* on PC3 Gene Expression

**Figure 3:**
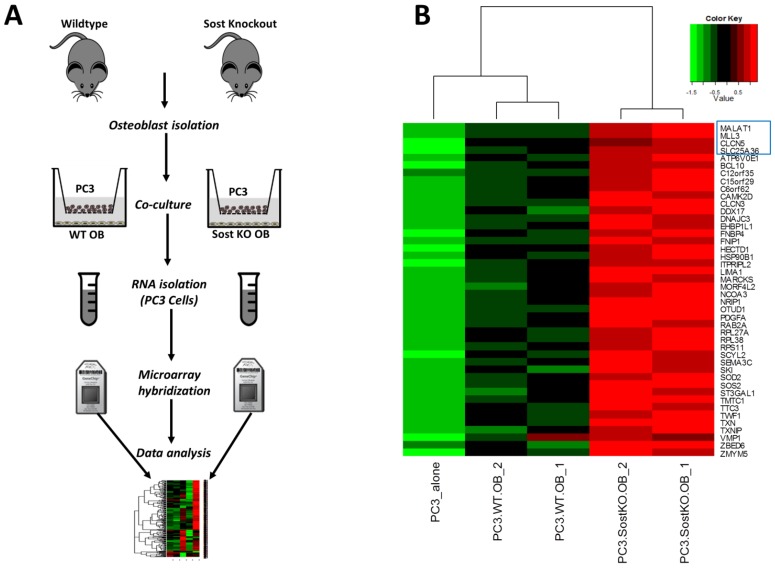
Co-culture with WT and *Sost*^KO^ osteoblasts (OB) elicited different transcriptional changes in PC3 cells. (**A**) OBs were isolated and co-cultured with PC3 cells in transwells. 48 h post-plating RNA was isolated and analyzed using microarrays; (**B**) Heat map showing genes up-regulated in both PC3-WT osteoblast (PC3.WT.OB) co-cultures compared to PC3 alone and PC3-*Sost*^KO^ osteoblast (PC3.SostKO.OB) co-cultures compared to PC3-WT osteoblasts. Individual samples are represented as columns and genes as rows. Four genes from this list, *MALAT1*, *CLCN5*, *MLL3* and *SLC25A36* (in blue rectangle), were found to be up-regulated in metastatic prostate cancer compared to clinically localized cancer.

Microarray data showed that the Wnt pathway inhibitor *Sost* was down-regulated in osteoblast co-cultures compared to osteoblast monoculture. We confirmed this observation using qPCR and found a ~3.5-fold reduction in *Sost* expression in osteoblast co-cultures compared to monocultures (Figure 1C). We also evaluated changes in Sost protein expression using immunocytochemistry and found a significant reduction in Sost expression in UMR cells co-cultured with PC3 cells compared to UMR cells cultured alone (Figure 1D). Wnt signaling has been shown to play a major role in regulating bone metabolism and loss of function of *Sost* leads to increased bone formation [27]. Disregulated Wnt signaling has also been shown to play a major role in cancer progression and metastasis [50,51,52,53]. This led us to hypothesize that elevated Wnt signaling in the cancer microenvironment due to reduced *Sost* expression may have a significant effect on prostate cancer metastasis. To understand how *Sost* levels in the bone microenvironment effect prostate cancer gene expression we co-cultured prostate cancer cells with primary osteoblasts purified from WT and *Sost*^KO^ calvaria and measured gene expression changes in the PC3 cells (Figure 3A).

Our results showed 88 genes up- and one gene down-regulated in response to altered *Sost* expression (Table S4). Of all the significantly up-regulated transcripts, probes corresponding to 44 transcripts were elevated greater than 1.5-fold in PC3-*WT* osteoblast co-cultures compared to PC3 monocultures (Figure 3B). Gene ontology analysis of differentially expressed genes revealed that lack of *Sost* in the bone microenvironment up-regulated several genes associated with the GO term “chemotaxis” including *MET*, *PDGFA*, *FER*, *NRP2*, and *EZR* in prostate cancer. Other enriched GO terms include: “cellular protein complex disassembly”, “actin filament organization”, “regulation of cell differentiation”, and “protein phosphorylation” (Table 3).

**Table 3:** Enriched gene ontology terms associated with genes differentially expressed between PC3-*Sost*^KO^ osteoblast co-cultures and PC3-WT osteoblast co-cultures.

GO ID	GO Term	Genes
GO:0043624	Cellular protein complex disassembly	*RPL27A*, *LIMA1*, *RPL37A*, *RPL38*, *MAP1B*, *EML4*, *RPS11*, *TWF1*
GO:0006468	Protein phosphorylation	*SCYL2*, *JAK1*, *MET*, *HSP90B1*, *BMPR2*, *PTPRA*, *PDGFA*, *SQSTM1*, *MAP4K5*, *CAMK2D*, *CLK4*, *FER*, *FNIP1*, *ST3GAL1*, *BCL10*, *GNAS*, *DNAJC3*, *TWF1*
GO:1902680	Positive regulation of RNA biosynthetic process	*NCOA2*, *MET*, *NCOA3*, *NRIP1*, *DDX17*, *ATRX*, *MORF4L2*, *SQSTM1*, *KAT6A*, *ELF1*, *E2F3*, *BCL10*, *MLLT10*, *SKI*, *NR1D2*
GO:0006935	Chemotaxis	*EZR*, *MET*, *WASL*, *SEMA3C*, *BMPR2*, *PTPRA*, *PDGFA*, *FER*, *NRP2*, *SOS2*
GO:0045595	Regulation of cell differentiation	*BHLHE41*, *MET*, *NCOA3*, *BMPR2*, *DDX17*, *PTPRA*, *MAP1B*, *MORF4L2*, *KAT6A*, *FNIP1*, *GNAS*, *SKI*, *TTC3*, *NR1D2*, *SOD2*
GO:0007015	Actin filament organization	*EZR*, *HSP90B1*, *WASL*, *LIMA1*, *FER*, *TWF1*

Next, we downloaded metastatic and primary prostate cancer gene expression data [54] from GEO (GSE3325) and identified genes differentially expressed between metastatic prostate cancer and localized primary prostate cancer. Subsequently, we compared genes differentially expressed between PC3 alone, PC3-WT osteoblast co-cultures, and PC3-*Sost*^KO^ osteoblast co-cultures to genes differentially expressed between primary prostate cancer and metastatic prostate cancer. Four genes in particular, *MALAT1*, *CLCN5*, *MLL3*, and *SLC25A36*, that were up-regulated in metastatic prostate cancer compared to primary prostate cancer were also displayed an enhanced expression in PC3-WT osteoblast co-cultures and PC3-*Sost*^KO^ osteoblast co-cultures compared to PC3 alone (Figure 3B).

**Figure 4:**
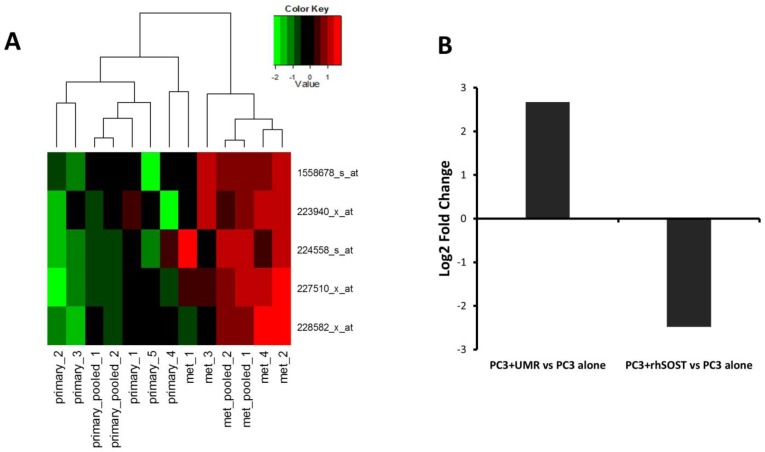
LncRNA *MALAT1* is transcriptionally modulated by Sost. (**A**) Heat map showing the expression values of *MALAT1* probes in primary prostate cancer samples (primary) and metastatic prostate cancer samples (met). Individual samples are represented as columns and *MALAT1* probes as rows; (**B**) *MALAT1* was ~6-fold up-regulated in prostate cancer cells co-cultured with UMR osteoblastic cells and ~5.6-fold down-regulated in PC3 cells treated with recombinant human SOST (rhSOST) compared to prostate cancer monocultures, as confirmed by qPCR.

*MALAT1* is an lncRNA previously shown to promote proliferation and invasion of many cancers, including prostate cancer [55,56]. We found *MALAT1* ~4 fold up-regulated in PC3 cells co-cultured with WT osteoblasts. Lack of *Sost* in osteoblasts further enhanced *MALAT1* expression (Figure 3B). Up-regulation of *MALAT1* in metastatic prostate cancer (Figure 4A) and PC3 cells co-cultured with *Sost*^KO^ osteoblasts (Figure 3B) suggests that reduced *Sost* expression in the bone microenvironment may promote prostate cancer metastasis at least in part through the up-regulation of non-coding RNA *MALAT1*.

### 3.4. Sost Is a Regulator of MALAT1 in Prostate Cancer Cells

Microarray revealed that *MALAT1* was up-regulated in prostate cancer cells co-cultured with WT osteoblasts compared to PC3 alone, suggesting that factors secreted by osteoblasts up-regulate *MALAT1*. Subsequently, we co-cultured PC3 cells with UMR osteoblasts and, using qPCR, confirmed that the factors secreted by osteoblasts up-regulated *MALAT1* expression in prostate cancer. The qPCR data showed a ~6-fold increase in *MALAT1* expression in PC3 cells co-cultured with UMR osteoblasts compared to PC3 monocultures (Figure 4B). To test whether *Sost* is a regulator of *MALAT1* in prostate cancer we cultured PC3 cells with recombinant human SOST for 48 h and quantified *MALAT1* expression using qPCR. Treatment with recombinant SOST resulted in ~5.6-fold reduction in *MALAT1* expression (Figure 4B), suggesting that *Sost* in the tumor microenvironment may have an inhibitory effect on *MALAT1* and down-regulation of *Sost* in the bone microenvironment may enhance *MALAT1* expression in prostate cancer.

## 4. Discussion

Previous studies have shown that cancer cells secrete factors that affect growth and differentiation of bone cells [2,3] and that the bone microenvironment secretes factors that promote cancer metastasis to bone [2,3]. In this study, we identified several genes including the cytokines *Il-6*, *Tgfb2*, *Cxcl1*, and *Ctgf*, metallopeptidases *Mmp13*, *Adamts1*, *Adamts4*, and *Adamts5*, and transcription factors *Junb*, *Fos*, *Stat3* and *Cebpb* that were up-regulated in osteoblasts as a result of prostate cancer–osteoblast interactions. Many of these genes have previously been shown to promote cancer metastasis and/or bone remodeling [29,30,31,32,33,36,37,38,39,47,48,49]. Our findings suggest that factors secreted by metastasizing prostate cancer alter osteoblastic gene expression and change the bone microenvironment in its own favor. In addition, we also show that the expression of the Wnt inhibitor *Sost* is significantly down-regulated in osteoblasts co-cultured with prostate cancer cells, strongly suggesting that in addition to modulating bone metabolism, *Sost* may also play a role in cancer–bone interaction potentially facilitating cancer metastasis to bone. Co-cultures of PC3 cells with *Sost*^KO^ and WT osteoblasts revealed lncRNA *MALAT1* as one of the most highly up-regulated transcripts in PC3 cells co-cultured with *Sost*^KO^ osteoblasts, an RNA we also found elevated in clinical samples of metastatic prostate cancer.

*MALAT1*, also known as *NEAT2*, is a highly conserved lncRNA overexpressed in several human cancers and linked to enhanced cell proliferation, migration, and/or invasion of cancers, such as: prostate [55], breast [57], lung [58], liver [59], esophageal [60,61], pancreatic [62], gastric [62], colorectal [63], bladder [64], cervical [65], and osteosarcoma [66]. Knocking down *MALAT1* by siRNA in prostate cancer cell lines inhibited cell growth, invasion, and migration and induced cell cycle arrest in the G0/G1 phases [55]. These results indicate that *MALAT1* has a pro-proliferative and migratory role in prostate cancer. *MALAT1* has been shown to regulate tumor progression and metastasis by modulating different signaling pathways, including the Snail pathway in colon cancer [67], PI3K/Akt pathway in osteosarcoma cells [66], and ERK/MAPK pathway in gallbladder cancer cells [68]. It has also been suggested that *MALAT1* regulates splicing of pre-mRNAs by modulating SR splicing factors [69] and controls cell cycle progression and cell death by modulating transcription factors *P53*, *E2F1*, and *B*-*MYB* [70]. Here, we found *MALAT1*’s expression to be regulated by *Sost*, suggesting that altered *Sost* expression in bone microenvironment may regulate prostate cancer proliferation, migration, or invasion at least in part by modulating *MALAT1* expression in prostate cancer. Further studies will be required to elucidate the molecular mechanism by which *MALAT1* regulates prostate cancer metastasis.

In summary, we identified several potential regulators of prostate cancer bone metastasis, including the Wnt inhibitor *Sost*, that were differentially regulated in osteoblasts as a result of osteoblast-prostate cancer interactions. We also identified osteoblastic *Sost* as a major regulator of PC3 gene expression and identified lncRNA *MALAT1* as a target gene regulated by *Sost* in prostate cancer cells. Further clarification of the mechanisms by which Sost/Wnt/MALAT1 pathway regulate prostate cancer metastasis may open up new avenues of therapeutic intervention in treating prostate cancer metastasis to bone.

## Supplementary Files

Supplementary File 1

## References

[1] Siegel R., Naishadham D., Jemal A. (2013). Cancer statistics, 2013. CA Cancer J. Clin..

[2] Tantivejkul K., Kalikin L.M., Pienta K.J. (2004). Dynamic process of prostate cancer metastasis to bone. J. Cell Biochem..

[3] Krzeszinski J.Y., Wan Y. (2015). New therapeutic targets for cancer bone metastasis. Trends Pharmacol. Sci..

[4] Logothetis C.J., Lin S.H. (2005). Osteoblasts in prostate cancer metastasis to bone. Nat. Rev. Cancer.

[5] Morrison C., Mancini S., Cipollone J., Kappelhoff R., Roskelley C., Overall C. (2011). Microarray and proteomic analysis of breast cancer cell and osteoblast co-cultures: Role of osteoblast matrix metalloproteinase (MMP)-13 in bone metastasis. J. Biol. Chem..

[6] Florencio-Silva R., Sasso G.R., Sasso-Cerri E., Simoes M.J., Cerri P.S. (2015). Biology of bone tissue: Structure, function, and factors that influence bone cells. BioMed Res. Int..

[7] Giunciuglio D., Cai T., Filanti C., Manduca P., Albini A. (1995). Effect of osteoblast supernatants on cancer cell migration and invasion. Cancer Lett..

[8] Krishnan V., Shuman L.A., Sosnoski D.M., Dhurjati R., Vogler E.A., Mastro A.M. (2011). Dynamic interaction between breast cancer cells and osteoblastic tissue: Comparison of two- and three-dimensional cultures. J. Cell Physiol..

[9] Rajski M., Vogel B., Baty F., Rochlitz C., Buess M. (2012). Global gene expression analysis of the interaction between cancer cells and osteoblasts to predict bone metastasis in breast cancer. PLoS ONE.

[10] Yang J., Fizazi K., Peleg S., Sikes C.R., Raymond A.K., Jamal N., Hu M., Olive M., Martinez L.A., Wood C.G. (2001). Prostate cancer cells induce osteoblast differentiation through a *Cbfa1*-dependent pathway. Cancer Res..

[11] Collette N.M., Genetos D.C., Economides A.N., Xie L., Shahnazari M., Yao W., Lane N.E., Harland R.M., Loots G.G. (2012). Targeted deletion of *Sost* distal enhancer increases bone formation and bone mass. Proc. Natl. Acad. Sci. USA.

[12] Bellows C.G., Sodek J., Yao K.L., Aubin J.E. (1986). Phenotypic differences in subclones and long-term cultures of clonally derived rat bone cell lines. J. Cell Biochem..

[13] Gentleman R.C., Carey V.J., Bates D.M., Bolstad B., Dettling M., Dudoit S., Ellis B., Gautier L., Ge Y., Gentry J. (2004). Bioconductor: Open software development for computational biology and bioinformatics. Genome Biol..

[14] Irizarry R.A., Hobbs B., Collin F., Beazer-Barclay Y.D., Antonellis K.J., Scherf U., Speed T.P. (2003). Exploration, normalization, and summaries of high density oligonucleotide array probe level data. Biostatistics.

[15] Smyth G.K. (2004). Linear models and empirical bayes methods for assessing differential expression in microarray experiments. Stat. Appl. Genet. Mol. Biol..

[16] Chen J., Bardes E.E., Aronow B.J., Jegga A.G. (2009). Toppgene suite for gene list enrichment analysis and candidate gene prioritization. Nucleic Acids Res..

[17] Montojo J., Zuberi K., Rodriguez H., Bader G.D., Morris Q. (2014). Genemania: Fast gene network construction and function prediction for cytoscape. F1000Research.

[18] Shannon P., Markiel A., Ozier O., Baliga N.S., Wang J.T., Ramage D., Amin N., Schwikowski B., Ideker T. (2003). Cytoscape: A software environment for integrated models of biomolecular interaction networks. Genome Res..

[19] Livak K.J., Schmittgen T.D. (2001). Analysis of relative gene expression data using real-time quantitative PCR and the 2^−∆∆*C*t^ method. Methods.

[20] Frara N., Abdelmagid S.M., Sondag G.R., Moussa F.M., Yingling V.R., Owen T.A., Popoff S.N., Barbe M.F., Safadi F.F. (2015). Transgenic expression of osteoactivin/gpnmb enhances bone formation *in vivo* and osteoprogenitor differentiation *ex vivo*. J. Cell Physiol..

[21] Brun J., Fromigue O., Dieudonne F.X., Marty C., Chen J., Dahan J., Wei Y., Marie P.J. (2013). The LIM-only protein FHL2 controls mesenchymal cell osteogenic differentiation and bone formation through Wnt5a and Wnt10b. Bone.

[22] Julien M., Khoshniat S., Lacreusette A., Gatius M., Bozec A., Wagner E.F., Wittrant Y., Masson M., Weiss P., Beck L. (2009). Phosphate-dependent regulation of MGP in osteoblasts: Role of ERK1/2 and Fra-1. J. Bone Miner. Res..

[23] Mackenzie N.C., Huesa C., Rutsch F., MacRae V.E. (2012). New insights into NPP1 function: Lessons from clinical and animal studies. Bone.

[24] Rowe P.S. (2015). A unified model for bone-renal mineral and energy metabolism. Curr. Opin. Pharmacol..

[25] Casimiro S., Guise T.A., Chirgwin J. (2009). The critical role of the bone microenvironment in cancer metastases. Mol. Cell Endocrinol..

[26] Bonfil R.D., Chinni S., Fridman R., Kim H.R., Cher M.L. (2007). Proteases, growth factors, chemokines, and the microenvironment in prostate cancer bone metastasis. Urol. Oncol..

[27] Honasoge M., Rao A.D., Rao S.D. (2014). Sclerostin: Recent advances and clinical implications. Curr. Opin. Endocrinol. Diabetes Obes..

[28] Ellies D.L., Economou A., Viviano B., Rey J.P., Paine-Saunders S., Krumlauf R., Saunders S. (2014). *Wise* regulates bone deposition through genetic interactions with *Lrp5*. PLoS ONE.

[29] Tawara K., Oxford J.T., Jorcyk C.L. (2011). Clinical significance of interleukin (IL)-6 in cancer metastasis to bone: Potential of anti-IL-6 therapies. Cancer Manag. Res..

[30] Vindrieux D., Escobar P., Lazennec G. (2009). Emerging roles of chemokines in prostate cancer. Endocr. Relat. Cancer.

[31] Hardaway A.L., Herroon M.K., Rajagurubandara E., Podgorski I. (2015). Marrow adipocyte-derived CXCL1 and CXCL2 contribute to osteolysis in metastatic prostate cancer. Clin. Exp. Metastasis.

[32] Buijs J.T., Stayrook K.R., Guise T.A. (2011). TGF-β in the bone microenvironment: Role in breast cancer metastases. Cancer Microenviron..

[33] Yang F., Tuxhorn J.A., Ressler S.J., McAlhany S.J., Dang T.D., Rowley D.R. (2005). Stromal expression of connective tissue growth factor promotes angiogenesis and prostate cancer tumorigenesis. Cancer Res..

[34] Lin Y.H., Tian Y., Wang J.S., Jiang Y.G., Luo Y., Chen Y.T. (2015). Pituitary tumor-transforming gene 1 regulates invasion of prostate cancer cells through MMP13. Tumour Biol..

[35] Mendonsa A.M., VanSaun M.N., Ustione A., Piston D.W., Fingleton B.M., Gorden D.L. (2015). Host and tumor derived MMP13 regulate extravasation and establishment of colorectal metastases in the liver. Mol. Cancer.

[36] Mochizuki S., Okada Y. (2007). Adams in cancer cell proliferation and progression. Cancer Sci..

[37] Ishimi Y., Miyaura C., Jin C.H., Akatsu T., Abe E., Nakamura Y., Yamaguchi A., Yoshiki S., Matsuda T., Hirano T. (1990). IL-6 is produced by osteoblasts and induces bone resorption. J. Immunol..

[38] Onan D., Allan E.H., Quinn J.M., Gooi J.H., Pompolo S., Sims N.A., Gillespie M.T., Martin T.J. (2009). The chemokine *CXCL1* is a novel target gene of parathyroid hormone (PTH)/PTH-related protein in committed osteoblasts. Endocrinology.

[39] Mundy G.R., Boyce B., Hughes D., Wright K., Bonewald L., Dallas S., Harris S., Ghosh-Choudhury N., Chen D., Dunstan C. (1995). The effects of cytokines and growth factors on osteoblastic cells. Bone.

[40] Arnott J.A., Lambi A.G., Mundy C., Hendesi H., Pixley R.A., Owen T.A., Safadi F.F., Popoff S.N. (2011). The role of connective tissue growth factor (CTGF/CCN2) in skeletogenesis. Crit. Rev. Eukaryot. Gene Expr..

[41] Tang S.Y., Herber R.P., Ho S.P., Alliston T. (2012). Matrix metalloproteinase-13 is required for osteocytic perilacunar remodeling and maintains bone fracture resistance. J. Bone Miner. Res..

[42] Zhao L., Huang J., Guo R., Wang Y., Chen D., Xing L. (2010). Smurf1 inhibits mesenchymal stem cell proliferation and differentiation into osteoblasts through JunB degradation. J. Bone Miner. Res..

[43] Kenner L., Hoebertz A., Beil T., Keon N., Karreth F., Eferl R., Scheuch H., Szremska A., Amling M., Schorpp-Kistner M. (2004). Mice lacking JunB are osteopenic due to cell-autonomous osteoblast and osteoclast defects. J. Cell Biol..

[44] Henriquez B., Hepp M., Merino P., Sepulveda H., van Wijnen A.J., Lian J.B., Stein G.S., Stein J.L., Montecino M. (2011). C/EBPβ binds the P1 promoter of the *Runx2* gene and up-regulates Runx2 transcription in osteoblastic cells. J. Cell Physiol..

[45] Itoh S., Udagawa N., Takahashi N., Yoshitake F., Narita H., Ebisu S., Ishihara K. (2006). A critical role for interleukin-6 family-mediated Stat3 activation in osteoblast differentiation and bone formation. Bone.

[46] Zhang C., Tang W., Li Y. (2012). Matrix metalloproteinase 13 (MMP13) is a direct target of osteoblast-specific transcription factor osterix (Osx) in osteoblasts. PLoS ONE.

[47] Nannuru K.C., Futakuchi M., Varney M.L., Vincent T.M., Marcusson E.G., Singh R.K. (2010). Matrix metalloproteinase (MMP)-13 regulates mammary tumor-induced osteolysis by activating MMP9 and transforming growth factor-β signaling at the tumor-bone interface. Cancer Res..

[48] Shah M., Huang D., Blick T., Connor A., Reiter L.A., Hardink J.R., Lynch C.C., Waltham M., Thompson E.W. (2012). An MMP13-selective inhibitor delays primary tumor growth and the onset of tumor-associated osteolytic lesions in experimental models of breast cancer. PLoS ONE.

[49] Akech J., Wixted J.J., Bedard K., van der Deen M., Hussain S., Guise T.A., van Wijnen A.J., Stein J.L., Languino L.R., Altieri D.C. (2010). Runx2 association with progression of prostate cancer in patients: Mechanisms mediating bone osteolysis and osteoblastic metastatic lesions. Oncogene.

[50] Sottnik J.L., Hall C.L., Zhang J., Keller E.T. (2012). Wnt and Wnt inhibitors in bone metastasis. BoneKEy Rep..

[51] Rabbani S.A., Arakelian A., Farookhi R. (2013). LRP5 knockdown: Effect on prostate cancer invasion growth and skeletal metastasis *in vitro* and *in vivo*. Cancer Med..

[52] Tai H.C., Chang A.C., Yu H.J., Huang C.Y., Tsai Y.C., Lai Y.W., Sun H.L., Tang C.H., Wang S.W. (2014). Osteoblast-derived Wnt-induced secreted protein 1 increases VCAM-1 expression and enhances prostate cancer metastasis by down-regulating miR-126. Oncotarget.

[53] Yang X., Li L., Huang Q., Xu W., Cai X., Zhang J., Yan W., Song D., Liu T., Zhou W. (2015). Wnt signaling through Snail1 and Zeb1 regulates bone metastasis in lung cancer. Am. J. Cancer Res..

[54] Varambally S., Yu J., Laxman B., Rhodes D.R., Mehra R., Tomlins S.A., Shah R.B., Chandran U., Monzon F.A., Becich M.J. (2005). Integrative genomic and proteomic analysis of prostate cancer reveals signatures of metastatic progression. Cancer Cell.

[55] Ren S., Liu Y., Xu W., Sun Y., Lu J., Wang F., Wei M., Shen J., Hou J., Gao X. (2013). Long noncoding RNA MALAT-1 is a new potential therapeutic target for castration resistant prostate cancer. J. Urol..

[56] Gutschner T., Hammerle M., Diederichs S. (2013). *MALAT1*—A paradigm for long noncoding RNA function in cancer. J. Mol. Med..

[57] Zhao Z., Chen C., Liu Y., Wu C. (2014). 17β-Estradiol treatment inhibits breast cell proliferation, migration and invasion by decreasing MALAT-1 RNA level. Biochem. Biophys. Res. Commun..

[58] Tano K., Mizuno R., Okada T., Rakwal R., Shibato J., Masuo Y., Ijiri K., Akimitsu N. (2010). MALAT-1 enhances cell motility of lung adenocarcinoma cells by influencing the expression of motility-related genes. FEBS Lett..

[59] Lai M.C., Yang Z., Zhou L., Zhu Q.Q., Xie H.Y., Zhang F., Wu L.M., Chen L.M., Zheng S.S. (2012). Long non-coding RNA *MALAT-1* overexpression predicts tumor recurrence of hepatocellular carcinoma after liver transplantation. Med. Oncol..

[60] Hu L., Wu Y., Tan D., Meng H., Wang K., Bai Y., Yang K. (2015). Up-regulation of long noncoding RNA MALAT1 contributes to proliferation and metastasis in esophageal squamous cell carcinoma. J. Exp. Clin. Cancer Res. CR.

[61] Wang X., Li M., Wang Z., Han S., Tang X., Ge Y., Zhou L., Zhou C., Yuan Q., Yang M. (2015). Silencing of long noncoding RNA *MALAT1* by miR-101 and miR-217 inhibits proliferation, migration, and invasion of esophageal squamous cell carcinoma cells. J. Biol. Chem..

[62] Jiao F., Hu H., Yuan C., Wang L., Jiang W., Jin Z., Guo Z. (2014). Elevated expression level of long noncoding RNA *MALAT-1* facilitates cell growth, migration and invasion in pancreatic cancer. Oncol. Rep..

[63] Xu C., Yang M., Tian J., Wang X., Li Z. (2011). MALAT-1: A long non-coding RNA and its important 3′ end functional motif in colorectal cancer metastasis. Int. J. Oncol..

[64] Ying L., Chen Q., Wang Y., Zhou Z., Huang Y., Qiu F. (2012). Upregulated *MALAT-1* contributes to bladder cancer cell migration by inducing epithelial-to-mesenchymal transition. Mol. BioSyst..

[65] Guo F., Li Y., Liu Y., Wang J., Li G. (2010). Inhibition of metastasis-associated lung adenocarcinoma transcript 1 in CaSki human cervical cancer cells suppresses cell proliferation and invasion. Acta Biochim. Biophys. Sin..

[66] Dong Y., Liang G., Yuan B., Yang C., Gao R., Zhou X. (2015). MALAT1 promotes the proliferation and metastasis of osteosarcoma cells by activating the PI3K/Akt pathway. Tumour Biol..

[67] Kan J.Y., Wu D.C., Yu F.J., Wu C.Y., Ho Y.W., Chiu Y.J., Jian S.F., Hung J.Y., Wang J.Y., Kuo P.L. (2015). Chemokine (C–C motif) ligand 5 is involved in tumor-associated dendritic cell-mediated colon cancer progression through non-coding RNA MALAT-1. J. Cell Physiol..

[68] Wu X.S., Wang X.A., Wu W.G., Hu Y.P., Li M.L., Ding Q., Weng H., Shu Y.J., Liu T.Y., Jiang L. (2014). MALAT1 promotes the proliferation and metastasis of gallbladder cancer cells by activating the ERK/MAPK pathway. Cancer Biol. Ther..

[69] Tripathi V., Ellis J.D., Shen Z., Song D.Y., Pan Q., Watt A.T., Freier S.M., Bennett C.F., Sharma A., Bubulya P.A. (2010). The nuclear-retained noncoding RNA MALAT1 regulates alternative splicing by modulating SR splicing factor phosphorylation. Mol. Cell.

[70] Tripathi V., Shen Z., Chakraborty A., Giri S., Freier S.M., Wu X., Zhang Y., Gorospe M., Prasanth S.G., Lal A. (2013). Long noncoding RNA MALAT1 controls cell cycle progression by regulating the expression of oncogenic transcription factor B-MYB. PLoS Genet..

