# Supplementary material for: Cancer–Osteoblast Interaction Reduces Sost Expression in Osteoblasts and Up-Regulates lncRNA MALAT1 in Prostate Cancer
Source: Microarrays (Basel). 2015 Oct 29;4(4):503–19. doi: 10.3390/microarrays4040503 (PMC4996404; doi:10.3390/microarrays4040503)
Supplement: Supplementary File 1 [file microarrays-04-00503-s001.pdf]

Supplementary Information

Table S1. Primers used for quantitative PCR.

Species	Gene	Forward	Reverse
Rat	*Il-6*	TCCATCTGCCCTTCAGGAAC	ATACTGGTCTGTTGTGGGTGG
Rat	*Ctgf*	CTGCCAAAGATGGTGCACC	ATTTCCCGGGCAGCTTGAC
Rat	*Adamts1*	TGACACAGCGATTCTGTTCACC	CCAATTCATGGGCTGTGGTG
Rat	*Sost*	GCCTTCGTTGCTGTGGAGAG	GCTGTACTCGGACACGTCTTTG
Rat	*Sostdc1*	GCAGCAACAGCACCTTGAATC	TGTATTTGGTGGACCGCAGTTC
Rat	*Mmp13*	GCCTTCAAGGTCTGGTCTGATG	AAAGCGTGTGCCAGAAGACC
Human	*MALAT1*	GCTGGGGGAAGTTAAATATG	GTTAATGTCAGCCCAGTAGG

Table S2. Genes more than two-fold up- or down-regulated in UMR-106 osteoblastic cells co-cultured with prostate cancer cells (PC3) compared to UMR-106 cells cultured alone.

Gene	Log2 Fold Change	*p*-Value	Adjusted *p*-Value
Mmp13	7.379026511	4.19×10^{-7}	0.00268023
Gng5	7.223894682	9.97×10^{-5}	0.019744835
Hspb11	6.154948595	9.11×10^{-5}	0.019528593
Ifitm1	4.876494699	1.04×10^{-6}	0.00268023
Cp	4.788420215	9.15×10^{-7}	0.00268023
Cav1	4.710019852	1.61×10^{-6}	0.003570654
Lbp	4.110706375	4.47×10^{-6}	0.005777101
Steap4	4.092986124	6.32×10^{-6}	0.007010559
Sec61a1	3.924895985	0.000636	0.04167131
Wisp2	3.856685884	3.63×10^{-5}	0.015103966
Cp	3.751837407	3.87×10^{-6}	0.005777101
Slpi	3.635567198	5.15×10^{-6}	0.006144527
Igfbp5	3.620444597	4.46×10^{-6}	0.005777101
A2m	3.471647174	2.17×10^{-5}	0.011604771
Cp	3.43628021	4.14×10^{-6}	0.015531321
Utp11l	3.434692273	0.000289	0.030120265
Il1rl1	3.407958196	9.57×10^{-5}	0.019553503
Kcnma1	3.341138681	0.000927	0.049112152
Lcp2	3.032559949	4.96×10^{-5}	0.015531321
Igfbp5	3.005322318	1.75×10^{-5}	0.010463506
Lcp2	2.96408364	0.000129	0.021140106
Sephs1	2.823095157	0.000508	0.037927329
Il6	2.807731249	2.96×10^{-5}	0.013108396
Egr2	2.781177887	1.94×10^{-5}	0.010885868
Pacrg	2.766776364	2.47×10^{-5}	0.011906394
Cxcl1	2.551449698	6.76×10^{-5}	0.017496943
Mgp	2.518383482	0.000517	0.038072221
Cebpd	2.446527999	4.74×10^{-5}	0.015531321
LOC690768	2.424213769	4.98×10^{-5}	0.015531321
Egr1	2.391719762	8.01×10^{-5}	0.019430575
Maf	2.328651566	0.000137	0.021691694

Table S2. *Cont.*

Gene	Log2 Fold Change	*p*-Value	Adjusted *p*-Value
Fam20c	2.28896788	3.8×10^{-5}	0.015103966
Sfrp4	2.283767959	7.43×10^{-5}	0.018309318
Tubb4b	2.269699129	4.67×10^{-5}	0.015531321
Itgb3	2.248692653	0.000106	0.019744835
Fst	2.160638095	0.000527	0.03813544
Nr4a1	2.159699899	0.000193	0.025661861
LOC685067	2.130158298	0.000157	0.023351615
Milr1	2.126473716	0.000287	0.030120265
Tbx18	2.04560636	0.000199	0.025958606
Adamts4	2.043578289	0.000354	0.032508229
Sostdc1	1.968348717	9.18×10^{-5}	0.019528593
Slfn3	1.94161152	0.000654	0.04248144
Slc1a3	1.936053744	0.000119	0.019860957
Junb	1.933333388	0.000417	0.034999079
LOC365985	1.850302469	0.000265	0.029012693
Rarb	1.779069222	0.000162	0.023351615
Sbno2	1.76970024	0.00016	0.023351615
Cpne8	1.747024502	0.000668	0.043011784
Pecam1	1.746836203	0.000141	0.0221523
Fos	1.745639593	0.00012	0.019860957
Lum	1.745462682	0.00012	0.019860957
Enpp3	1.745181674	0.000318	0.031052101
Sned1	1.732815148	0.000328	0.031458772
Ddx60	1.730363161	0.000381	0.033707214
LOC290595	1.714683948	0.000162	0.023351615
Dhrs3	1.711017884	0.000155	0.023351615
Btg2	1.663826772	0.000411	0.034939252
Tmem176a	1.649114303	0.0002	0.025958606
Etv1	1.648364577	0.000492	0.037927329
Adamts1	1.639779418	0.000357	0.032508229
Rgs2	1.63895833	0.000161	0.023351615
Rtp4	1.631378976	0.000159	0.023351615
Gpnmb	1.621638758	0.00023	0.027913204
Zfat	1.586637322	0.000201	0.025958606
Gpx3	1.586520436	0.000182	0.024498747
Ifitm3	1.584473394	0.000348	0.032365926
Tmcc3	1.572494999	0.00044	0.03593996
RT1-S3	1.568845855	0.000947	0.049648639
Igsf3	1.561877664	0.00025	0.028747279
Ddx58	1.560208302	0.000313	0.030924625
Igsf3	1.552538981	0.000255	0.028747279
Etv1	1.549842194	0.000246	0.028747279
Mafb	1.529663777	0.000222	0.027732607
Col13a1	1.518290628	0.00045	0.036532692
Dusp6	1.515067995	0.000609	0.040777546

Table S2. *Cont.*

Gene	Log2 Fold Change	*p*-Value	Adjusted *p*-Value
Adamts5	1.514558749	0.000499	0.037927329
Thbd	1.489286539	0.000251	0.028747279
Etv1	1.483179838	0.000719	0.043760523
Nfkbiz	1.474853457	0.000318	0.031052101
Irf1	1.474435736	0.00029	0.030120265
C1s	1.470117495	0.000271	0.029197214
RGD1309362	1.454792995	0.000609	0.040777546
Fndc1	1.453980928	0.000407	0.034939252
Igfbp4	1.451800078	0.000301	0.030717545
Irx3	1.448141648	0.000275	0.029391049
Spry4	1.443735586	0.000387	0.033785709
Clec2d	1.442610656	0.000798	0.045688605
Stat3	1.441311554	0.000385	0.033727008
Col3a1	1.435325652	0.000503	0.037927329
Col14a1	1.426672404	0.000714	0.043760523
Clec11a	1.424848014	0.00031	0.030885177
Mgst1	1.40354156	0.00035	0.032365926
Limch1	1.396031082	0.000517	0.038072221
Limch1	1.389008755	0.000886	0.048251424
Errfi1	1.369208762	0.000673	0.043011784
Pdgfra	1.368405638	0.000599	0.040566672
Jag1	1.366861206	0.000616	0.041055327
Maff	1.366701205	0.000468	0.037033815
Zfp36	1.361923861	0.000502	0.037927329
Ecm1	1.352201495	0.000673	0.043011784
Cldn10	1.337597617	0.000546	0.038689123
Pde4b	1.333246022	0.000814	0.046125666
RT1-S3	1.328608388	0.000794	0.045688605
Enpp1	1.328111803	0.000434	0.035642023
Fhl2	1.316993183	0.000766	0.045560588
C1qtnf1	1.307716459	0.000763	0.045560588
Iqca1	1.305180645	0.000453	0.036532692
Ctgf	1.299752782	0.000518	0.038072221
Etv4	1.267451271	0.000544	0.038689123
Etv1	1.262528701	0.00061	0.040777546
Tmem178	1.251635087	0.000525	0.03813544
Scara5	1.236160074	0.000769	0.045560588
Zfp36l1	1.231278015	0.000686	0.043011784
Smpd1	1.228495811	0.000585	0.04016975
Rgl1	1.218917979	0.000634	0.04167131
Cebpb	1.21173545	0.000687	0.043011784
Tgfb2	1.193588498	0.000752	0.0454512
Dusp1	1.188385752	0.000907	0.048546529
Vwa3b	1.162353304	0.000762	0.045560588

Table S2. *Cont.*

Gene	Log2 Fold Change	*p*-Value	Adjusted *p*-Value
Ier2	1.159718751	0.000923	0.049112152
Prkar1b	1.140146444	0.000848	0.046985366
Ggt1	1.114173778	0.000956	0.049980306
Vwa3b	1.162353304	0.000762	0.045560588
Ier2	1.159718751	0.000923	0.049112152
Prkar1b	1.140146444	0.000848	0.046985366
Ggt1	1.114173778	0.000956	0.049980306
Ednrb	−1.148889398	0.000785	0.045688605
Gucy1a3	−1.152896202	0.000927	0.049112152
Cgref1	−1.173553696	0.000713	0.043760523
Rtn4rl1	−1.182717896	0.000695	0.043155284
Larp1b	−1.1895208	0.000881	0.04815378
Atp1a2	−1.193219176	0.0009	0.048468148
Hhex	−1.194112413	0.000778	0.045688605
Tesc	−1.19528509	0.000717	0.043760523
Ddah1	−1.212222992	0.000813	0.046125666
Ntrk1	−1.234078116	0.000581	0.04016975
Ass1	−1.271680635	0.000801	0.045688605
Adarb1	−1.276598466	0.000846	0.046985366
Rab11fip4	−1.280019914	0.000568	0.039722703
Pls1	−1.316352215	0.00042	0.035050623
Cryab	−1.321516965	0.000526	0.03813544
Ppp1r1b	−1.353793377	0.000395	0.034214469
Nudt11	−1.36185788	0.000682	0.043011784
Bcl11b	−1.372178396	0.000376	0.033532672
Gatm	−1.376250722	0.000422	0.035050623
Mylpf	−1.38181054	0.000358	0.032508229
Csgalnact1	−1.386079167	0.000365	0.032747997
Fam195a	−1.390335429	0.000326	0.031426385
Phgdh	−1.39423251	0.000799	0.045688605
Psat1	−1.408704291	0.000432	0.035642023
Pcdh20	−1.417212857	0.00088	0.04815378
Bik	−1.428538912	0.000382	0.033707214
LOC100360867	−1.430099998	0.00094	0.049449833
Efhc2	−1.451958035	0.000342	0.03217657
Gjb5	−1.47141358	0.000528	0.03813544
Pcsk6	−1.48512512	0.000508	0.037927329
Fam189a2	−1.506543161	0.00031	0.030885177
Ppp1r1a	−1.54174931	0.000773	0.045596975
Apln	−1.562663696	0.000238	0.028213947
Fam198b	−1.608505146	0.000259	0.028899645
Car3	−1.612191512	0.000177	0.02408535
Nup210	−1.614280857	0.000297	0.030567541
Cd200	−1.632823121	0.000191	0.025593021
Slc13a5	−1.696584071	0.00032	0.031052101

Table S2. *Cont.*

Gene	Log2 Fold Change	*p*-Value	Adjusted *p*-Value
Ppm1l	−1.710561103	0.000583	0.04016975
Nrip3	−1.716346808	0.000133	0.021359606
Bmp3	−1.722220439	0.000154	0.023351615
Cd55	−1.738180076	0.000172	0.023688918
Vsnl1	−1.757975259	0.00022	0.027732607
Nexn	−1.778536045	0.00082	0.046125666
Mpp4	−1.792569128	0.000148	0.022963175
Atp2b2	−1.809843285	0.000635	0.04167131
Pcdh20	−1.813859613	0.000101	0.019744835
Hrc	−1.818929277	0.000227	0.027747838
Atp2b2	−1.831779646	0.000221	0.027732607
Metrnl	−1.848975604	0.000112	0.019744835
Cacna2d3	−1.919424858	0.000902	0.048468148
Blnk	−1.96511831	0.000112	0.019744835
Chac1	−2.09858954	8.97×10^{-5}	0.019528593
Bcl11b	−2.101680223	8.81×10^{-5}	0.019528593
Bmper	−2.128139935	4.8×10^{-5}	0.015531321
Slc36a2	−2.307489216	4.54×10^{-5}	0.015531321
Phex	−2.365544258	3.04×10^{-5}	0.013108396
Slc8a3	−2.420870879	8.27×10^{-5}	0.019528593
Myl1	−2.519145881	2.48×10^{-5}	0.011906394
Sox6	−2.526611686	0.000119	0.019860957
Sost	−2.829981741	3.71×10^{-5}	0.015103966
Mamdc2	−2.858764991	8.55×10^{-5}	0.019528593
Ckmt2	−3.01695192	1.59×10^{-5}	0.010463506
Panx3	−3.19516858	4.73×10^{-5}	0.015531321
Vsnl1	−3.269760826	8.7×10^{-6}	0.008435516
Phospho1	−3.481812101	1.32×10^{-5}	0.009286334
Smpd3	−4.175122995	6.36×10^{-5}	0.017496943

Table S3. Top 200 GO (gene ontology) terms associated with genes more than two-fold up- or down-regulated in UMR-106 osteoblastic cells co-cultured with prostate cancer cells (PC3) compared to UMR-106 cells cultured alone.

GO ID	GO Term	No. of Genes	Adjusted *p*-Value
GO:0009888	tissue development	58	2.74×10^{-15}
GO:0001503	ossification	25	9.23×10^{-12}
GO:0009611	response to wounding	41	3.29×10^{-10}
GO:0009719	response to endogenous stimulus	43	8.10×10^{-10}
GO:0010243	response to organonitrogen compound	30	7.58×10^{-9}
GO:1901698	response to nitrogen compound	31	9.29×10^{-9}
GO:0050793	regulation of developmental process	47	4.40×10^{-8}
GO:0030278	regulation of ossification	15	5.04×10^{-8}
GO:0009612	response to mechanical stimulus	15	7.59×10^{-8}
GO:1901700	response to oxygen-containing compound	37	1.30×10^{-7}

Table S3. *Cont.*

GO ID	GO Term	No. of Genes	Adjusted *p*-Value
GO:0072359	circulatory system development	30	1.55×10^{-7}
GO:0072358	cardiovascular system development	30	1.55×10^{-7}
GO:2000026	regulation of multicellular organismal development	39	1.66×10^{-7}
GO:0014070	response to organic cyclic compound	27	2.38×10^{-7}
GO:0001501	skeletal system development	21	2.82×10^{-7}
GO:0045595	regulation of cell differentiation	37	5.14×10^{-7}
GO:0051248	negative regulation of protein metabolic process	24	6.07×10^{-7}
GO:0030097	hemopoiesis	26	8.16×10^{-7}
GO:0061061	muscle structure development	22	1.13×10^{-6}
GO:0001933	negative regulation of protein phosphorylation	16	1.13×10^{-6}
GO:0008283	cell proliferation	43	1.19×10^{-6}
GO:0060537	muscle tissue development	18	1.26×10^{-6}
GO:0006955	immune response	36	1.55×10^{-6}
GO:0042326	negative regulation of phosphorylation	17	1.61×10^{-6}
GO:0048534	hematopoietic or lymphoid organ development	26	1.61×10^{-6}
GO:0008285	negative regulation of cell proliferation	23	1.77×10^{-6}
GO:0009887	organ morphogenesis	28	1.80×10^{-6}
GO:0042127	regulation of cell proliferation	36	1.92×10^{-6}
GO:0045936	negative regulation of phosphate metabolic process	18	1.92×10^{-6}
GO:0010563	negative regulation of phosphorus metabolic process	18	1.92×10^{-6}
GO:0014706	striated muscle tissue development	17	2.78×10^{-6}
GO:0006469	negative regulation of protein kinase activity	13	3.10×10^{-6}
GO:0002520	immune system development	26	3.36×10^{-6}
GO:0009725	response to hormone	26	7.36×10^{-6}
GO:0033673	negative regulation of kinase activity	13	9.14×10^{-6}
GO:0001944	vasculature development	21	9.14×10^{-6}
GO:0006357	regulation of transcription from RNA polymerase II promoter	37	1.08×10^{-5}
GO:0007167	enzyme linked receptor protein signaling pathway	28	1.32×10^{-5}
GO:0032269	negative regulation of cellular protein metabolic process	20	1.32×10^{-5}
GO:0010605	negative regulation of macromolecule metabolic process	38	1.32×10^{-5}
GO:0009891	positive regulation of biosynthetic process	36	1.32×10^{-5}
GO:0006366	transcription from RNA polymerase II promoter	39	1.32×10^{-5}
GO:0006952	defense response	35	1.47×10^{-5}
GO:0001775	cell activation	26	1.52×10^{-5}

Table S3. *Cont.*

GO ID	GO Term	No. of Genes	Adjusted *p*-Value
GO:0060429	epithelium development	29	1.56×10^{-5}
GO:0051241	negative regulation of multicellular organismal process	17	1.57×10^{-5}
GO:0061448	connective tissue development	13	1.57×10^{-5}
GO:0031214	biomineral tissue development	10	1.97×10^{-5}
GO:0010628	positive regulation of gene expression	32	2.03×10^{-5}
GO:0071310	cellular response to organic substance	39	2.05×10^{-5}
GO:0007596	blood coagulation	19	2.08×10^{-5}
GO:0030282	bone mineralization	9	2.08×10^{-5}
GO:0050817	coagulation	19	2.21×10^{-5}
GO:0051173	positive regulation of nitrogen compound metabolic process	34	2.21×10^{-5}
GO:0007599	hemostasis	19	2.25×10^{-5}
GO:0002682	regulation of immune system process	30	2.37×10^{-5}
GO:0043086	negative regulation of catalytic activity	23	2.72×10^{-5}
GO:0009892	negative regulation of metabolic process	39	3.06×10^{-5}
GO:0033993	response to lipid	22	3.13×10^{-5}
GO:0050878	regulation of body fluid levels	21	3.87×10^{-5}
GO:0045944	positive regulation of transcription from RNA polymerase II promoter	25	4.51×10^{-5}
GO:0048585	negative regulation of response to stimulus	28	4.56×10^{-5}
GO:0001568	blood vessel development	19	4.72×10^{-5}
GO:0031328	positive regulation of cellular biosynthetic process	34	4.85×10^{-5}
GO:0007517	muscle organ development	15	6.54×10^{-5}
GO:0010557	positive regulation of macromolecule biosynthetic process	32	7.81×10^{-5}
GO:0009628	response to abiotic stimulus	26	8.39×10^{-5}
GO:1903034	regulation of response to wounding	15	8.92×10^{-5}
GO:0045935	positive regulation of nucleobase-containing compound metabolic process	32	8.92×10^{-5}
GO:0031400	negative regulation of protein modification process	16	9.28×10^{-5}
GO:0051591	response to cAMP	8	1.35×10^{-4}
GO:0048514	blood vessel morphogenesis	17	1.35×10^{-4}
GO:0042060	wound healing	20	1.59×10^{-4}
GO:0051348	negative regulation of transferase activity	13	1.59×10^{-4}
GO:0035295	tube development	19	1.59×10^{-4}
GO:0071495	cellular response to endogenous stimulus	25	1.61×10^{-4}
GO:0031324	negative regulation of cellular metabolic process	35	1.63×10^{-4}
GO:0051254	positive regulation of RNA metabolic process	29	1.71×10^{-4}

Table S3. *Cont.*

GO ID	GO Term	No. of Genes	Adjusted *p*-Value
GO:0045893	positive regulation of transcription, DNA-templated	28	1.91×10^{-4}
GO:0043588	skin development	14	1.95×10^{-4}
GO:0002521	leukocyte differentiation	16	1.95×10^{-4}
GO:0050673	epithelial cell proliferation	13	2.42×10^{-4}
GO:0008544	epidermis development	13	2.42×10^{-4}
GO:0051216	cartilage development	10	2.54×10^{-4}
GO:0042325	regulation of phosphorylation	28	2.68×10^{-4}
GO:0044092	negative regulation of molecular function	24	2.69×10^{-4}
GO:0014074	response to purine-containing compound	9	2.69×10^{-4}
GO:0000165	MAPK cascade	19	2.69×10^{-4}
GO:0048732	gland development	15	2.69×10^{-4}
GO:0051093	negative regulation of developmental process	20	2.74×10^{-4}
GO:0006954	inflammatory response	18	3.00×10^{-4}
GO:1902680	positive regulation of RNA biosynthetic process	28	3.05×10^{-4}
GO:0048545	response to steroid hormone	14	3.43×10^{-4}
GO:0030198	extracellular matrix organization	14	3.81×10^{-4}
GO:0043062	extracellular structure organization	14	3.88×10^{-4}
GO:0071901	negative regulation of protein serine/threonine kinase activity	8	4.27×10^{-4}
GO:0006873	cellular ion homeostasis	16	4.42×10^{-4}
GO:0048646	anatomical structure formation involved in morphogenesis	24	4.42×10^{-4}
GO:0040011	locomotion	31	4.82×10^{-4}
GO:0006936	muscle contraction	12	5.18×10^{-4}
GO:0023014	signal transduction by protein phosphorylation	19	5.44×10^{-4}
GO:1901652	response to peptide	15	5.54×10^{-4}
GO:0006875	cellular metal ion homeostasis	15	5.77×10^{-4}
GO:0035051	cardiocyte differentiation	8	5.93×10^{-4}
GO:0001649	osteoblast differentiation	10	5.93×10^{-4}
GO:1901701	cellular response to oxygen-containing compound	21	5.93×10^{-4}
GO:0003012	muscle system process	13	5.93×10^{-4}
GO:0046683	response to organophosphorus	8	6.46×10^{-4}
GO:0045669	positive regulation of osteoblast differentiation	6	6.60×10^{-4}
GO:0050801	ion homeostasis	18	7.07×10^{-4}
GO:0055065	metal ion homeostasis	16	7.07×10^{-4}
GO:0051094	positive regulation of developmental process	22	7.29×10^{-4}
GO:0010035	response to inorganic substance	14	7.29×10^{-4}
GO:0051270	regulation of cellular component movement	18	7.29×10^{-4}
GO:0051480	cytosolic calcium ion homeostasis	11	7.35×10^{-4}
GO:0006939	smooth muscle contraction	7	7.35×10^{-4}

Table S3. *Cont.*

GO ID	GO Term	No. of Genes	Adjusted *p*-Value
GO:0016310	phosphorylation	36	7.35×10^{-4}
GO:0007507	heart development	15	7.88×10^{-4}
GO:0070372	regulation of ERK1 and ERK2 cascade	9	8.48×10^{-4}
GO:0035914	skeletal muscle cell differentiation	6	8.55×10^{-4}
GO:0048584	positive regulation of response to stimulus	31	8.57×10^{-4}
GO:0010942	positive regulation of cell death	15	8.80×10^{-4}
GO:0048705	skeletal system morphogenesis	10	8.86×10^{-4}
GO:0051240	positive regulation of multicellular organismal process	18	8.86×10^{-4}
GO:0009968	negative regulation of signal transduction	22	8.94×10^{-4}
GO:0080134	regulation of response to stress	23	9.59×10^{-4}
GO:0030334	regulation of cell migration	16	9.59×10^{-4}
GO:0045597	positive regulation of cell differentiation	18	9.59×10^{-4}
GO:0050776	regulation of immune response	20	9.59×10^{-4}
GO:0090257	regulation of muscle system process	9	9.59×10^{-4}
GO:0007519	skeletal muscle tissue development	9	9.59×10^{-4}
GO:0030003	cellular cation homeostasis	15	9.59×10^{-4}
GO:0060087	relaxation of vascular smooth muscle	3	9.62×10^{-4}
GO:0072012	glomerulus vasculature development	4	1.01×10^{-3}
GO:0045444	fat cell differentiation	9	1.02×10^{-3}
GO:0007178	transmembrane receptor protein serine/threonine kinase signaling pathway	12	1.05×10^{-3}
GO:0043434	response to peptide hormone	14	1.06×10^{-3}
GO:0006937	regulation of muscle contraction	8	1.07×10^{-3}
GO:0044057	regulation of system process	14	1.07×10^{-3}
GO:0060047	heart contraction	9	1.07×10^{-3}
GO:0001932	regulation of protein phosphorylation	23	1.07×10^{-3}
GO:0070848	response to growth factor	18	1.07×10^{-3}
GO:0003015	heart process	9	1.09×10^{-3}
GO:0045932	negative regulation of muscle contraction	4	1.12×10^{-3}
GO:0030879	mammary gland development	8	1.12×10^{-3}
GO:0006928	movement of cell or subcellular component	32	1.14×10^{-3}
GO:0043589	skin morphogenesis	5	1.14×10^{-3}
GO:0060538	skeletal muscle organ development	9	1.14×10^{-3}
GO:0030168	platelet activation	10	1.15×10^{-3}
GO:0043407	negative regulation of MAP kinase activity	6	1.21×10^{-3}
GO:0032101	regulation of response to external stimulus	17	1.22×10^{-3}
GO:0044557	relaxation of smooth muscle	3	1.25×10^{-3}
GO:0061440	kidney vasculature development	4	1.25×10^{-3}
GO:0090075	relaxation of muscle	4	1.25×10^{-3}
GO:0061437	renal system vasculature development	4	1.25×10^{-3}
GO:0045596	negative regulation of cell differentiation	16	1.26×10^{-3}

Table S3. *Cont.*

GO ID	GO Term	No. of Genes	Adjusted *p*-Value
GO:0070371	ERK1 and ERK2 cascade	9	1.27×10^{-3}
GO:1901607	alpha-amino acid biosynthetic process	6	1.33×10^{-3}
GO:0040012	regulation of locomotion	17	1.43×10^{-3}
GO:0045667	regulation of osteoblast differentiation	7	1.44×10^{-3}
GO:0043408	regulation of MAPK cascade	16	1.44×10^{-3}
GO:0022414	reproductive process	26	1.45×10^{-3}
GO:0023057	negative regulation of signaling	22	1.46×10^{-3}
GO:2000145	regulation of cell motility	16	1.46×10^{-3}
GO:0055080	cation homeostasis	16	1.46×10^{-3}
GO:0010648	negative regulation of cell communication	22	1.49×10^{-3}
GO:0006953	acute-phase response	5	1.53×10^{-3}
GO:0010544	negative regulation of platelet activation	3	1.61×10^{-3}
GO:0042592	homeostatic process	28	1.62×10^{-3}
GO:0030855	epithelial cell differentiation	16	1.73×10^{-3}
GO:0008016	regulation of heart contraction	8	1.75×10^{-3}
GO:0001525	angiogenesis	13	1.81×10^{-3}
GO:0048771	tissue remodeling	8	1.89×10^{-3}
GO:0071417	cellular response to organonitrogen compound	14	1.89×10^{-3}
GO:0016477	cell migration	23	1.89×10^{-3}
GO:0051707	response to other organism	18	1.91×10^{-3}
GO:0043207	response to external biotic stimulus	18	1.91×10^{-3}
GO:0071363	cellular response to growth factor stimulus	17	2.07×10^{-3}
GO:0022612	gland morphogenesis	7	2.10×10^{-3}
GO:0055082	cellular chemical homeostasis	16	2.18×10^{-3}
GO:0006940	regulation of smooth muscle contraction	5	2.26×10^{-3}
GO:0045682	regulation of epidermis development	5	2.45×10^{-3}
GO:0043200	response to amino acid	6	2.60×10^{-3}
GO:0031069	hair follicle morphogenesis	4	2.60×10^{-3}
GO:0022617	extracellular matrix disassembly	7	2.62×10^{-3}
GO:0061035	regulation of cartilage development	5	2.62×10^{-3}
GO:0060350	endochondral bone morphogenesis	5	2.83×10^{-3}
GO:0007610	behavior	17	2.85×10^{-3}
GO:0050727	regulation of inflammatory response	10	2.85×10^{-3}
GO:0001958	endochondral ossification	4	2.85×10^{-3}
GO:0036075	replacement ossification	4	2.85×10^{-3}
GO:0030509	BMP signaling pathway	7	2.91×10^{-3}
GO:0050678	regulation of epithelial cell proliferation	10	2.91×10^{-3}
GO:0060349	bone morphogenesis	6	2.95×10^{-3}
GO:0060443	mammary gland morphogenesis	5	2.95×10^{-3}
GO:0031347	regulation of defense response	15	3.01×10^{-3}
GO:0008015	blood circulation	13	3.01×10^{-3}
GO:0009607	response to biotic stimulus	18	3.01×10^{-3}
GO:0002252	immune effector process	16	3.08×10^{-3}
GO:0003013	circulatory system process	13	3.11×10^{-3}

Table S4. Genes more than two-fold up- or down-regulated in PC3 cells co-cultured with *Sost* ^{KO} osteoblasts compared to PC3 cells co-cultured with WT (wildtype) osteoblasts.

Genes	Log2 Fold Change	*p*-Value	Adjusted *p*-Value
RPL37A	3.7984	1.63×10^{-5}	0.0345
UBN2	3.6835	5.79×10^{-5}	0.0345
MALAT1	3.2281	0.000116	0.0394
SLC25A36	3.0704	1.09×10^{-5}	0.0345
HSP90B1	3.0685	5.24×10^{-5}	0.0345
DDX17	3.0633	0.000165	0.0419
EEF1D	2.9534	7.11×10^{-5}	0.0345
C12orf35	2.9452	1.67×10^{-5}	0.0345
GNAS	2.9232	1.39×10^{-5}	0.0345
RPL38	2.9194	9.45×10^{-6}	0.0345
ZBED6	2.8119	0.000214	0.0474
ZNF800	2.7524	1.96×10^{-5}	0.0345
MAP4K5	2.7270	6.85×10^{-5}	0.0345
ATP13A3	2.7142	0.0002	0.0466
ATP6V0E1	2.6892	2.76×10^{-5}	0.0345
MLL3	2.6759	3.91×10^{-5}	0.0345
EZR	2.6645	0.000141	0.0396
CLK4	2.6628	3.73×10^{-5}	0.0345
RAB3B	2.6213	6.89×10^{-5}	0.0345
SOD2	2.5752	5.64×10^{-5}	0.0345
TWF1	2.5484	1.71×10^{-5}	0.0345
VMP1	2.5188	0.00012	0.0394
ST3GAL1	2.5174	0.000104	0.0384
PDGFA	2.5096	3.31×10^{-5}	0.0345
PHLDB2	2.4801	7.95×10^{-5}	0.0345
EML4	2.4770	2.34×10^{-5}	0.0345
MARCKS	2.4571	1.97×10^{-5}	0.0345
MLL	2.4461	0.000104	0.0384
YIF1B	2.4248	5.72×10^{-5}	0.0345
WASL	2.3879	5.02×10^{-5}	0.0345
NR1D2	2.3861	2.78×10^{-5}	0.0345
TMTC1	2.3856	5.71×10^{-5}	0.0345
SEMA3C	2.3774	0.000112	0.0394
ZMYM2	2.3748	0.000102	0.0384
CLCN5	2.3682	4.65×10^{-5}	0.0345
ZNF652	2.3558	6.75×10^{-5}	0.0345
ITPRIPL2	2.3522	5.73×10^{-5}	0.0345
U2SURP	2.3430	7.42×10^{-5}	0.0345
RABGAP1L	2.3301	2.98×10^{-5}	0.0345
MET	2.3207	0.000133	0.0394
NCOA2	2.3204	4.23×10^{-5}	0.0345
RPS11	2.3178	2.74×10^{-5}	0.0345
MORF4L2	2.3127	6.54×10^{-5}	0.0345

Table S4. *Cont.*

Genes	Log2 Fold Change	*p*-Value	Adjusted *p*-Value
LIMA1	2.2990	4.83×10^{-5}	0.0345
HECTD1	2.2884	9.10×10^{-5}	0.0374
TXNIP	2.2771	0.000107	0.0384
ACBD3	2.2585	4.26×10^{-5}	0.0345
C6orf106	2.2570	0.000101	0.0384
TOX4	2.2316	7.68×10^{-5}	0.0345
SKI	2.2306	0.000159	0.0413
TBRG1	2.2298	0.000124	0.0394
CLCN3	2.2256	0.000101	0.0384
ELF1	2.2198	6.31×10^{-5}	0.0345
FER	2.2117	3.80×10^{-5}	0.0345
NCOA3	2.2112	0.000213	0.0474
DNAJC3	2.1636	4.68×10^{-5}	0.0345
OTUD1	2.1633	4.08×10^{-5}	0.0345
MLLT10	2.1401	9.99×10^{-5}	0.0384
ATRX	2.1244	0.000127	0.0394
NRIP1	2.1065	8.98×10^{-5}	0.0374
C15orf29	2.1014	0.000235	0.0496
SOS2	2.0970	0.000107	0.0384
HEATR7A	2.0965	0.000116	0.0394
AHNAK2	2.0915	0.000185	0.0436
FNIP1	2.0777	6.94×10^{-5}	0.0345
JAK1	2.0276	6.28×10^{-5}	0.0345
NRP2	2.0256	0.000129	0.0394
BCL10	2.0232	0.00018	0.0429
KAT6A	2.0174	0.000116	0.0394
PTPRA	2.0173	0.000169	0.0419
RPL27A	2.0162	0.000122	0.0394
TTC3	1.9963	0.00015	0.0399
TM4SF1	1.9630	0.00012	0.0394
LPP	1.9246	8.74×10^{-5}	0.0373
EHBP1L1	1.9128	0.000132	0.0394
E2F3	1.9071	0.000154	0.0405
CAMK2D	1.8964	0.000119	0.0394
BHLHE41	1.7969	0.000215	0.0474
C6orf62	1.7833	0.000221	0.0479
SCYL2	1.7793	0.000202	0.0466
FNBP4	1.7791	0.000239	0.0496
ZMYM5	1.7655	0.000165	0.0419
SQSTM1	1.7595	0.000208	0.0473
RANBP2	1.7464	0.00018	0.0429
RAB2A	1.7448	0.000177	0.0429
TXN	1.7151	0.00021	0.0474
BMPR2	1.7040	0.00024	0.0496
MAP1B	1.6790	0.00022	0.0479
LINC00326	−2.7521	7.28×10^{-5}	0.0345
